# Intestinal hypomotility in systemic sclerosis: a histological study into the sequence of events

**DOI:** 10.1007/s10067-020-05325-8

**Published:** 2020-08-18

**Authors:** M. den Braber-Ymker, M. C. Vonk, K. Grünberg, M. Lammens, I. D. Nagtegaal

**Affiliations:** 1 Department of Pathology 824, Radboud University Medical Center, PO Box 9101, 6500 HB Nijmegen, The Netherlands; 2 Department of Rheumatology, Radboud University Medical Center, Nijmegen, The Netherlands; 3 Department of Pathology, Antwerp University Hospital, University of Antwerp, Edegem, Belgium; 4 MIPRO, University of Antwerp, Antwerp, Belgium

**Keywords:** Histology, Intestinal dysmotility, Pathogenesis, Systemic sclerosis

## Abstract

**Objectives:**

The pathogenesis of intestinal involvement in systemic sclerosis (SSc) is thought to be a sequential process (vascular, neuronal, and consecutive muscular impairment), but understanding of the underlying histological changes and how they translate to symptoms, is still lacking. Therefore, we systematically investigated histological characteristics of SSc in the intestines, compared to controls.

**Methods:**

Autopsy material from the small bowel and colon was used for histological semiquantitative evaluation of the vasculature, enteric nervous system, interstitial cells of Cajal (ICC), and muscle layers, using a combination of histochemical and immunohistochemical stainings, according to guidelines of the Gastro 2009 International Working Group.

**Results:**

Vascular changes were most frequently encountered, represented by intima fibrosis in both arteries and small vessels, and represented by venous dilatation. Second, generalized fibrosis of the circular muscle layer was significantly more found in SSc patients than in controls. Third, reduction of submucosal nerve fibers and myenteric neurons was shown in the colon of four SSc patients, which may explain severe symptoms of intestinal dysmotility. The density of myenteric ICC network was decreased in the small bowel of SSc patients.

**Conclusions:**

The postulated sequential processes of intestinal involvement in SSc could not be supported by our histological evaluation. The interpatient diversity suggests that parallel processes occur, explaining the variety of histological features and clinical symptoms.

**Table:** 

**Key Points** *• Histological analysis showed vascular changes, fibrosis in the muscularis propria, and reduction of the ENS and ICC network in the intestines of SSc patients.* *• Pathophysiological mechanisms leading to intestinal dysmotility in SSc may be parallel rather than sequential.* *• The interpatient diversity suggests parallel pathophysiological processes, explaining the variety of histological features and clinical symptoms.*

**Electronic supplementary material:**

The online version of this article (10.1007/s10067-020-05325-8) contains supplementary material, which is available to authorized users.

## Introduction

Systemic sclerosis (SSc) is an autoimmune disease characterized by inflammation, vasculopathy, and fibrosis of the skin and internal organs. Dysfunction of the immune system may result in the production of autoantibodies [1, 2]. Although the major causes of death in SSc are pulmonary fibrosis, pulmonary hypertension, and cardiac disease, the most common organ complication is involvement of the gastrointestinal (GI) tract [1, 3, 4]. The esophagus is most commonly involved (up to 96% of patients), followed by the small intestine (40–88%) and colon (10–50%) [5, 6]. The development of GI symptoms is diverse, ranging from minimal complaints to significant dysmotility, leading to malnutrition, with wide variation in progression rates [7–9]. Most GI complications originate from decreased motility and have a major effect on the quality of life [7, 8]. In the small intestine, the major symptom is hypomotility, sometimes leading to pseudo-obstruction and bacterial overgrowth [10–12]. Manometric studies suggested that neurogenic and myogenic abnormalities may be the underlying cause [13–15]. Cholinergic neurotransmission may be inhibited by the presence of autoantibodies in these patients [16] and deposition of collagen in the small intestinal wall may result in pseudo-obstruction [11]. In the colon, hypomotility can result in delayed transit and constipation [11].

The development of intestinal dysmotility during SSc is currently incompletely understood. It has been hypothesized as a progressive sequence of events, starting with vascular damage. Subsequent neurogenic dysfunction may be the result from ischemia due to arteriolar changes in the vasa nervorum, compression by collagen deposition around the nerve fibers, or blocked cholinergic neurotransmission [1, 17]. Finally, the smooth muscle cells become affected, either as a direct result from ischemia or from neuronal damage. Damaged smooth muscle cells may result in atrophy of the muscle layers and fibrosis [11, 18, 19].

Systematic analysis of histological changes of the vasculature, neuronal structures, and smooth muscle may provide insight in the correlation of pathogenic processes in SSc, from which symptoms may be explained and a possible sequence of events may be deduced. Morphological features in the esophagus and stomach have been extensively described [20, 21]. In the esophagus, the major finding is smooth muscle atrophy, which may be preceded by loss of neural function, or may be a primary smooth muscle problem [20]. In the stomach, the main findings are fibrosis in the muscularis propria, accompanied by ultrastructural changes of smooth muscle cells and interstitial cells of Cajal (ICCs) [21]. In the intestines, no systematic histological study has been performed. Vascular changes have been described in the small arteries and capillaries of the intestines [19]. Some studies reported atrophy and fibrosis in the circular smooth muscle layer of the muscularis propria throughout the GI tract, including the small intestine and colon [17, 22]. Diffuse fibrosis in the longitudinal muscle layer and the serosa has been reported in the colon as well [17, 19].

In short, understanding of the underlying histological changes of the proposed sequential processes of intestinal dysmotility is still lacking. Hence, we systematically investigated the histological characteristics of SSc in the small intestine and colon, applying international guidelines for the study on motility disorders [23]. We focused on vasculature, the enteric nervous system (ENS), the myenteric network of ICCs, and the muscular layers.

## Methods

### Subjects

Archived autopsy formalin-fixed paraffin-embedded segments of the small bowel (*n* = 14) and colon (*n* = 18) were obtained from SSc patients without selection for dysmotility. Control autopsy material of the ileum (*n* = 22) and colon (*n* = 19) was obtained from non-SSc patients without intestinal motility problems or other relevant gastrointestinal comorbidities (according to the pathology reports), and considered as normal.

The local ethics committee approved the study (reference number 2014-1256). Samples were obtained in accordance with the Code of Conduct of the Federation of Medical Scientific Societies in the Netherlands [24].

### Tissue preparation

Sections were cut from formalin-fixed paraffin-embedded full-thickness tissue blocks for conventional histology or immunohistochemistry. Sections were deparaffinized, rehydrated in xylene and ethanol series, and rinsed in tap water by standard protocol.

### Histological staining

Sections of 4 μm were used for hematoxylin and eosin (H&E) and periodic acid Schiff (PAS) staining. Elastica van Gieson (EVG) staining was performed on 6-μm sections. Tissues were stained by standard protocols in a Medite TST 30 stainer (Klinipath, Duiven, the Netherlands).

Four-micrometer sections were used for Sirius Red staining. The slides were incubated with a 0.1% Picro-sirius red solution (Sirius red F3B dissolved in aqueous picric acid) for 1 h, washed in acetic acid water, dehydrated with ethanol, cleared with xylene, and mounted.

### Immunohistochemistry

Immunohistochemical staining was performed on 4-μm sections. Antibodies, suppliers, and dilutions are listed in Table 1.

**Table 1:** Primary antibodies used for immunohistochemistry

Antibody	Clone	Manufacturer	Dilution	Antigen retrieval
HuC/D	16A11	Molecular Probes	1:600	Sodium citrate 10 mM (pH 6.0) 30 min at 100 °C
S100	polyclonal	DAKO	1:10000	EDTA pH 9 10 min at 96 °C
CD117	YR145	Immunologic	1:200	None
α-smooth muscle actin (α-SMA)	1A4	Sigma	1:7500	None

For HuC/D staining, antigen retrieval was performed in sodium citrate (pH 6) at 100 °C for 30 min. Subsequently, endogenous peroxidase was blocked with 3% hydrogen peroxide in PBS for 20 min. Sections were then rinsed in PBS and incubated with primary antibody anti-HuC/D at 4 °C overnight. After washing in PBS, sections were incubated for 30 min with a secondary antibody (Powervision poly-HRP anti Ms./Rb/Rt IgG, Immunologic, Duiven, the Netherlands) at room temperature. Subsequently, sections were rinsed in PBS and immunoreactivity was developed with PowerDAB (Immunologic) for 7 min at room temperature. Sections were finally rinsed in tap water, counterstained with hematoxylin, rinsed in tap water, dehydrated in 100% ethanol and xylene, and mounted with Permount.

The other immunohistochemical staining reactions were performed in an automated LabVision Autostainer 480 (Klinipath, Duiven, the Netherlands). The method used for antigen retrieval depended on the antibody (Table 1). Subsequently, endogenous peroxidase was blocked with 3% hydrogen peroxide in methanol for 10 min. Sections were incubated with primary antibody for 60 min. Subsequently, the sections were incubated with Powervision poly-HRP anti Ms./Rb/Rt for 30 min, followed by staining with PowerDAB for 7 min and counterstaining with hematoxylin for 1 min. All incubations were performed at room temperature.

Tissue blocks containing different tissue types were used as controls, with known staining patterns for both positive and negative stained tissues.

### Microscopic analysis

Sections were evaluated blind to diagnosis. The histology of the bowel wall was examined by H&E. PAS was used to verify the presence or absence of polyglucosan inclusion bodies in the muscularis propria. The presence or absence of fibrosis was assessed in the submucosa, muscularis propria, and myenteric plexus on EVG stained sections, represented by focal accumulation of collagen in these parts of the bowel wall. Quantification of fibrosis in both layers of the muscularis propria was performed on Sirius Red stained sections using semi-automated image analysis.

The vasculature was evaluated on EVG stained sections. Intima fibrosis in submucosal arteries, veins, and small vessels was scored as absent or present. The presence of venous dilatation was analyzed and graded as absent, slight, moderate, or severe.

Immunohistochemically stained sections were assessed by semiquantitative scoring using visual analysis to evaluate systematically the neuronal structures and smooth muscle layers, as previously described [25]. The presence of neurons in ganglia was analyzed on HuC/D-stained sections. The number of neurons in relation to the present plexus was estimated in HuC/D sections as follows: 0, no neurons/low neuronal density and 1, high neuronal density. S100 was used to assess the distribution of nerve fibers (including nuclei of glial cells) in the submucosa, the myenteric plexus, and both muscle layers of the muscularis propria. The degree of distribution was scored as follows: 0, no/low density and 1, high density of positive fibers [25]. The network of ICCs surrounding the myenteric plexus was estimated on CD117-stained sections as described earlier [26]. The percentage of the circumference which is covered by CD117-positive cells was rated from 0 to 100% in 10% increments. Thus, a percentage of 0% represented no positive cells around the ganglia and in sections estimated as 100%; the ganglia were completely surrounded by CD117-positive cells. α-Smooth muscle actin (α-SMA) staining was used to assess the muscular layers. Staining intensities of circular and longitudinal muscle layers were classified in two grades: 0, no/weak and 1, strong staining intensity [25].

### Quantification of fibrosis

Sirius Red stained sections were scanned using a 3DHistech Pannoramic 250 Flash II scanner (3DHistech, Budapest, Hungary). Slides were digitized using a × 20 objective (resultant pixel resolution of 0.24 μm). A maximum of ten images of both the circular and longitudinal muscle layer per patient were taken at × 20 magnification using Pannoramic Viewer (3Dhistech), dependent on the size of the tissue fragment. Per image, the muscle area was manually annotated using a bright green–colored line. Staining artifacts and large blood vessels were excluded from the annotations. Subsequently, the images were analyzed using a modified version of the ImageJ macro developed by Hadi et al. [27], using Fiji (Fiji is Just ImageJ 1.47v, [28]). Our macro included the area within the bright green–demarcated annotation for analysis, while Hadi et al. excluded the area within the green annotation. Other minor changes in the Fiji macro were made to optimize the analysis for our tissue sections. The batch mode macro was used to analyze multiple images in a row. The modified batch mode macro is available as Supplementary material. The percentage of collagen (fibrosis) compared to the total amount of tissue within the green demarcated area in an image was quantified for the circular and longitudinal muscle layer, respectively.

### Differentiating focal and generalized fibrosis

The presence of fibrosis on EVG stained sections in circular and/or longitudinal layers was defined as “focal fibrosis.” The amount of collagen (Sirius Red) in the circular layer was represented as a percentage (Fig. 3c). We calculated 95% confidence intervals (CI) of means for each group: for SSc, lower CI were 48% (small bowel) and 47% (colon), and for controls, upper CI were 43% (small bowel) and 45% (colon). Consequently, we defined a percentage of collagen higher than 48% as “generalized fibrosis.”

### Statistical analysis

Categorical variables were described by percentages. Differences between categorical variables were assessed by the chi-square test (likelihood ratio, exact *p* values compared with the control group). Continuous variables were presented as means ± standard deviation (SD). The Mann-Whitney test was performed to compare the CD117 scores between groups. Wilcoxon signed-rank and McNemar tests were used for pairwise comparison of small bowel and colon tissues within one patient. In quantification of fibrosis, *T* tests were used to compare SSc and control groups, paired *T* tests for comparisons within one patient. A *p* value of 0.05 was considered significant. Data were analyzed by the IBM SPSS Statistics 22 Software (SPSS Inc., Chicago, IL, USA) and GraphPad Prism version 5.00 for Windows (GraphPad Software, San Diego California USA).

## Results

### Patients

Tissue material of 21 patients with SSc and 26 control patients was evaluated in this study. The SSc group consisted of 14 small bowel and 18 colon cases; from 11 patients, tissues of both locations were analyzed. In the control group, 22 small bowel cases were included, 19 colon cases, and of 15 patients both small bowel and colon tissues were evaluated. The mean age of the SSc group was 58 ± 12 years (38% male); in the control group, 66 ± 14 years (59% male). Information about SSc progression was obtained from the autopsy reports and patient dossiers. Twelve patients had the limited cutaneous form of SSc (lcSSc), nine patients the diffuse form of SSc (dcSSc) (Fig. 4b). Four patients reported clinical symptoms of severe gut dysmotility (e.g., pseudo-obstruction or need for total parenteral nutrition; Fig. 4b), 12 reported esophageal involvement, and seven reported cachexia (not explicitly related to hypomotility). Fourteen patients had proven lung disorder and four patients had proven renal involvement. No information was available about the type of autoantibodies. The duration of the disease was < 1 year in five patients, 1–5 years in eight patients, 6–10 years in four patients, 11–15 year in three patients, and unknown in 1 patient. Severe dysmotility in the colon may be related to a reduced ENS in some patients. No relationship could be found between the duration of the disease or other clinical characteristics and histological features.

### Vasculature

**Fig. 1:**
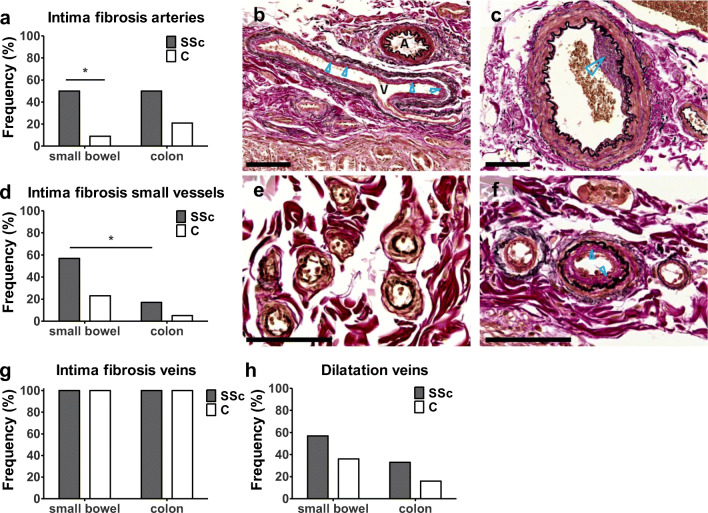
Histological findings in the vasculature. **a** Significantly more intima fibrosis was found in the submucosal arteries of the small bowel in SSc. **b** Shows a normal artery (*A*) and a vein (*V*) with intima fibrosis (pink band around lumen, *blue arrowheads*), EVG. **c** An artery with intima fibrosis (*blue arrowhead*), EVG. **d** Intima fibrosis in the small vessels was more frequently found in SSc patients compared to controls, not significant. Intima fibrosis was more present in small bowel than in colon. **e** Normal small vessels and **f** small vessels with intima fibrosis (pink band above internal elastical lamina, *blue arrowheads*), EVG. **g** Venous intima fibrosis was found in all control and SSc cases, a representative example is given in (b), indicated with “*V*.*”* **h** Dilatation of veins was more frequently observed in SSc, not significant. C, control; SSc, systemic sclerosis; EVG, elastic von Gieson. *p < 0.05 vs. control. Scale bars, 100 μm

Vascular changes tended to be more often present in patients with SSc compared to controls, as evident by trends present in all analyses. Only intima fibrosis in the submucosal arteries of the small bowel showed a significant difference (50% versus 9%; *p* = 0.014; Fig. 1). There were no differences between vascular abnormalities in small bowel and colon in SSc, except for the more frequent presence of intima fibrosis in the small vessels of the small bowel (*p* = 0.046).

### Enteric nervous system

**Fig. 2:**
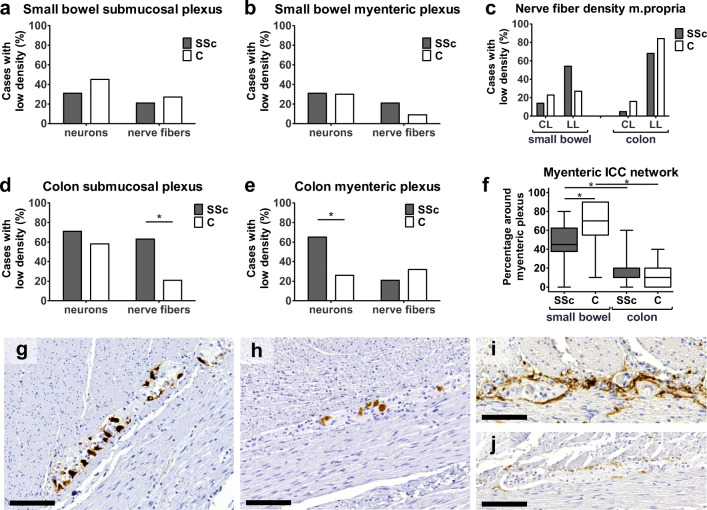
Histological findings in the enteric nervous system. **a** and **b** Show the percentage of cases with low density of neurons (HuC/D) and nerve fibers (S100) in the submucosal and myenteric plexus of small bowel, respectively. **c** The percentage of cases with a low S100-positive nerve fiber density in the muscularis propria (score 0) is shown. **d** and **e** Show the percentage of cases with low density of neurons and nerve fibers in the submucosal and myenteric plexus of colon, respectively. In the SSc group, the nerve fiber density is significantly decreased in the submucosal plexus (d) and the neuronal density is decreased in the myenteric plexus of colon (e). **f** The ICC network surrounding the myenteric plexus was significantly impaired in the small bowel in SSc. Representative examples of the HuC/D positive neurons in the myenteric plexus of a control sample **g** shows a higher neuronal density than that of a **h** SSc section. **i** A myenteric plexus of the small bowel is shown of a control that is completely surrounded by CD117 positive ICCs. **j** shows an example of the impaired myenteric ICC network in the small bowel in SSc. C, control; SSc, systemic sclerosis; CL, circular layer; LL, longitudinal layer; ICC, interstitial cell of Cajal. *p < 0.05. Scale bars, 100 μm

To evaluate the enteric nervous system we assessed both neuronal density and nerve fiber density, in particular in the submucosal plexus, the myenteric plexus and the muscularis propria. In the small bowel, no differences were observed between the SSc and the control group for the enteric nervous system (Fig. 2a–c). In the colon, neuronal density was decreased in the myenteric plexus (74% versus 35%, *p* = 0.042) of SSc patients, but not in the submucosal plexus (Fig. 2d). In contrast, the density of the submucosal nerve fiber network was frequently very low around the submucosal plexus of SSc patients (79% versus 37%; *p* = 0.020; Fig. 2d), but not in the myenteric plexus (Fig. 2e), nor in the muscularis propria (Fig. 2c).

### Myenteric ICC network

In the small bowel, significantly less CD117 positive cells were present around the circumference of the myenteric plexus in SSc, compared to the control group (33 ± 24% versus 65 ± 25%, *p* = 0.024). In the colon, the ICC network surrounding the myenteric plexus was comparable in both groups (15 ± 16% versus 12 ± 11%, *p* = 0.751; Fig. 2f). In both groups, CD117 scores were higher in the small bowel as compared to colon (*p* < 0.005).

### Muscularis propria

**Fig. 3:**
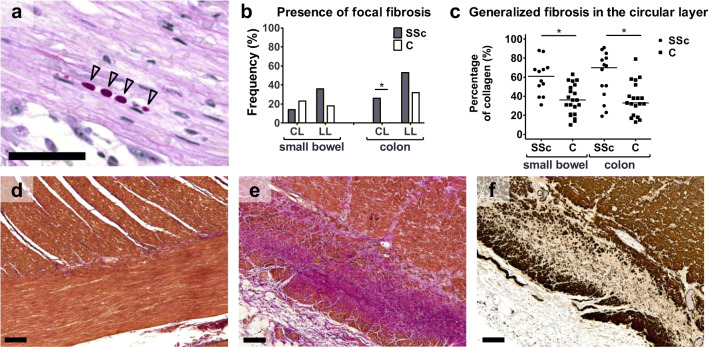
Histological findings in the muscularis propria. Polyglucosan inclusion bodies were more often present in the SSc group. **a** Shows an example of these inclusion bodies (*black arrowheads*)*.* Areas with remarkable fibrosis (excessive accumulation of collagen) were scored on EVG stained sections (c, d, e). **b** Shows the presence of focal fibrosis in the circular layer (CL) and longitudinal layer (LL) of the small bowel and colon, respectively. Fibrosis was significantly more frequently found in the CL of the colon in SSc patients (b). **c** The percentage of collagen was computed on Sirius Red stained sections, a percentage higher than 48% was defined as “generalized fibrosis.” Significantly more collagen was found in the circular layer in SSc patients compared to controls in both the small bowel and colon. **d** Shows the muscularis propria of the colon without fibrosis in a control section, EVG. **e** Excessive collagen accumulation (pink) in the LL of a colon section of SSc reveals fibrosis in, EVG. **f** In the same region, the absence of α-SMA staining is found. C, control; SSc, systemic sclerosis; EVG, elastic von Gieson; SMA, smooth muscle actin. *p < 0.05 Scale bars, 100 μm

To evaluate the muscularis propria, we investigated the smooth muscle cells (α-SMA immunohistochemistry), the presence of polyglucosan inclusion bodies, and the presence of fibrosis. No difference was present between SSc patients and controls with respect to the α-SMA filament proteins. Overall, more inclusion bodies were found in SSc patients compared to controls (25% versus 7%, *p* = 0.05; Fig. 3a). The presence of fibrosis was evaluated in both the circular and longitudinal muscle layer in two ways: presence of focal fibrosis was evaluated on EVG stained sections, while generalized fibrosis was evaluated on Sirius Red stained sections by quantification of the amount of collagen. Focal fibrosis is more frequently present in patients with SSc in particular in the circular layer of the colon (26% versus 0%, *p* = 0.046, Fig. 3b). Generalized fibrosis was also more often present in the circular layer of SSc patients (small bowel, 60 ± 18% (*n* = 12) versus 36 ± 16% (*n* = 21), *p* < 0.001; colon, 61 ± 25% (*n* = 14) versus 37 ± 18% (*n* = 19), *p* = 0.003; Fig. 3c). No differences between the SSc and control group were found in the longitudinal layer.

### Histological analysis of sequence of events in SSc

**Fig. 4:**
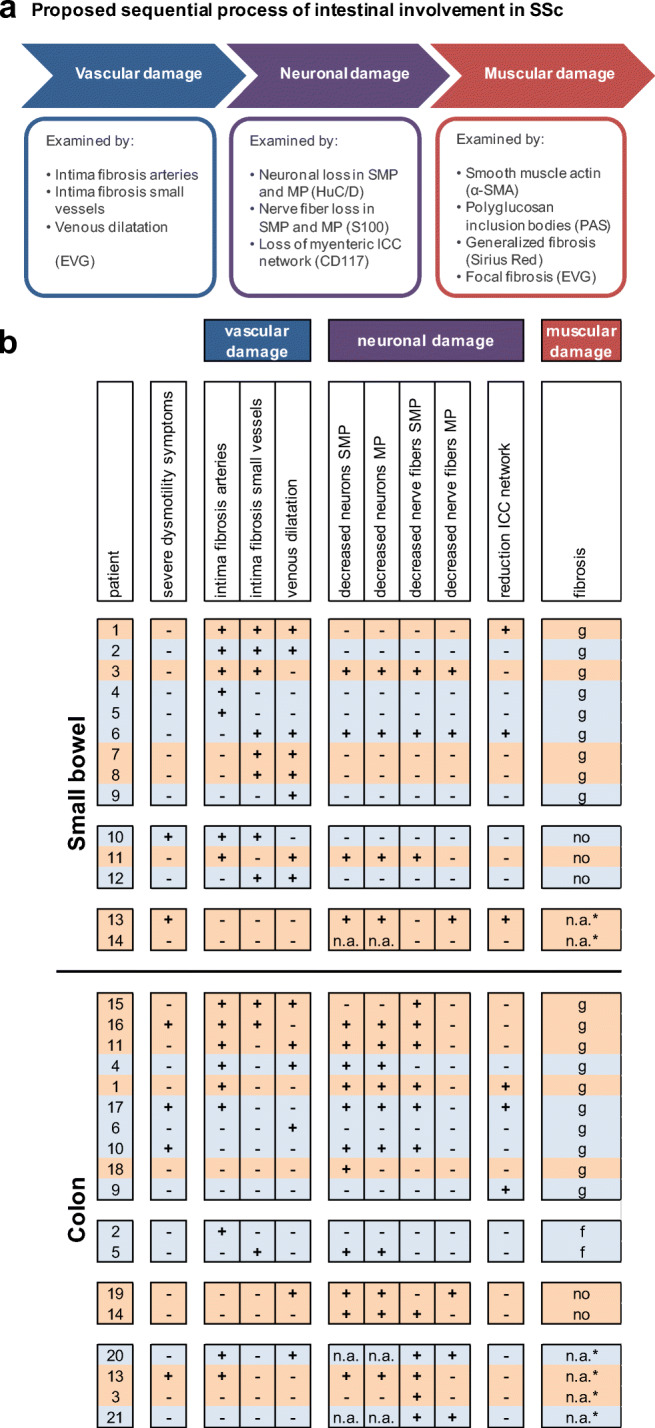
Evaluation of the proposed sequential pathogenesis of intestinal involvement in SSc. **a** Vascular, neuronal, and muscular damage were examined by several parameters. **b** An overview is given of each SSc patient, set against the presence of severe dysmotility symptoms, forms of vascular damage, neuronal damage, and muscular damage. The proposed end-stage of the disease is fibrosis of the muscularis propria. Three subgroups were distinguished: generalized fibrosis in the circular muscle layer (g), focal fibrosis (f), and no fibrosis. Each patient number correlates with one individual SSc patient. Orange color represents patients with limited cutaneous SSc, blue color patients with diffuse cutaneous SSc. *no focal fibrosis was present, but generalized fibrosis was not assessable. MP, myenteric plexus; SMP, submucosal plexus; ICC, interstitial cell of Cajal; n.a., not assessable

Fibrosis of the intestinal muscular layers is considered the end-stage of gastrointestinal SSc (Fig. 4a). Three subgroups were created: SSc patients with generalized fibrosis in the circular layer, SSc patients with focal fibrosis, and SSc patients with no relevant fibrosis in the muscle. Vascular and neural abnormalities were compared for these groups, but no differences could be observed (Fig. 4b).

In the small bowel, all SSc patients with and without fibrosis showed some form of vascular changes (i.e., intima fibrosis in arteries, in small vessels, and/or venous dilatation). In the colon, no vascular changes were observed in 3 of 10 patients with generalized fibrosis and in 1 of 2 patients without fibrosis. Vascular changes appeared to be more present in small bowel than in the colon. Changes in the ENS tended to be more frequently present in the colon than in the small intestine. In the colon, 4 of 7 patients with decreased neurons in both plexuses and a reduced nerve fiber network in the submucosal plexus reported symptoms of severe intestinal dysmotility. This association was not found in the small intestine. Patients with a low percentage of ICCs around the myenteric plexus did not present with symptoms of gut dysmotility. No differences between the lcSSc and dcSSc types were observed (Fig. 4b).

## Discussion

The histological features of SSc in the intestines were systematically investigated, focusing on vasculature, ENS, myenteric network of ICCs, and smooth muscle layers of the gut wall. The most frequent finding was vascular change in SSc patients in both the small bowel and colon, represented by intima fibrosis in the arteries and small vessels (particularly pronounced in small bowel), and venous dilatation. Vascular intima fibrosis has been described in other organs affected by SSc, including the esophagus and lungs [20, 29].

Second, remarkable generalized fibrosis was present in the circular layer of the small bowel and colon of SSc patients. SSc has been shown to commonly involve the circular muscle layer [17, 22]. Our findings confirm this and extend it by showing focal fibrosis being more frequently present in the longitudinal layers of the small bowel and colon of SSc patients. In the esophagus, Roberts et al. assessed the loss of muscle cells (defined as “smooth muscle atrophy”), and they defined thickening of the muscle layer as “fibrosis” [20]. The figures in their study show replacement of smooth muscle cells by collagen. We found collagen patterns similar to that in the esophagus, defined here as “fibrosis.” Polyglucosan inclusion bodies were significantly more frequently observed in SSc cases, which may be associated with intestinal myopathy [30]. There was no clear relationship between the presence of polyglucosan inclusions and symptoms of intestinal dysmotility in our study.

Third, we did not find changes of the ENS in the small bowel of SSc patients, but some significant differences between SSc patients and controls were shown in the colon: less nerve fibers were found in the submucosal plexus, and the neuronal density in the myenteric plexus was decreased. This may be related to symptoms of intestinal hypomotility. In addition, the ICC network around the myenteric plexus in the small bowel of SSc patients was reduced compared to the control group, which was not clearly associated with symptoms of dysmotility. Loss of ICCs can be observed as secondary to the absence of enteric neurons, for example, in Hirschsprung’s disease [31, 32] or to impaired extrinsic innervations, as is the case in spinal cord injury [25]. However, we did not find any correlation between decreased neuronal density and myenteric ICC scores in our study.

Comparison of small bowel and colon samples from each individual patient showed that intima fibrosis in the small vessels was more frequent in the small bowel, as was the presence of polyglucosan inclusion bodies. Clinically, up to 90% of SSc, patients have complaints from involvement of the small bowel including hypomotility, secondary bacterial overgrowth, and small bowel pseudo-obstruction [11]. An association between histological changes and these severe small intestinal complaints could not be found in our study. In the colon, more changes in the ENS were found compared to the small bowel. Furthermore, focal fibrosis of the muscularis propria was more pronounced in the colon than in the small bowel. However, the presence of generalized fibrosis in the circular muscle layer may have more effect on smooth muscle function than focal fibrosis. Reduction of the ENS may explain the symptoms of severe colonic dysmotility in four patients. Clinically, colon involvement is less frequent, and consists of delayed transit due to hypomotility [11].

Intestinal involvement in SSc has been proposed as a sequential process, which might start with vascular damage, followed by autonomic neuronal dysfunction, smooth muscle atrophy, and fibrosis [18, 19, 33]. However, the exact mechanisms are still poorly understood [1]. Since fibrosis of the muscularis propria is the considered end-stage lesion, we expected that in patients with fibrosis changes in the vasculature and ENS would be present as well (because we assume that these changes are not reversible). Case studies have indeed indicated that neuronal damage precedes smooth muscle atrophy and fibrosis in the gastrointestinal tract [17, 19]. However, in our larger series, we could find histological indications for this correlation only in part of SSc patients: vascular and neuronal changes were shown in small bowel in two of nine patients and in colon in six of ten patients with generalized fibrosis of the muscularis propria. Therefore, the pathogenic mechanisms of gut involvement may be more complex. Our results indicate that parallel processes could be involved in part of SSc patients rather than sequential processes. The initial event in the pathogenesis of intestinal SSc may be either autonomic axonal degeneration (sympathetic overactivity) resulting in vascular instability, autoimmune-related (e.g., antibodies against myenteric neurons), or a combination of both [34]. Manometric studies have shown evidence for a neuropathic phase preceding a myopathic phase in gut involvement [35, 36]. However, SSc patients may develop manometric abnormalities before the presence of histological changes [1], which may explain that we did not find histological neuropathic features in all patients with generalized fibrosis of the circular muscle layer. Results of manometry were not available in our study. On the other hand, smooth muscle function may directly be affected by different processes: tissue ischemia as result of vascular instability may trigger a repair reaction, leading to excessive collagen deposition [34]. Furthermore, antibodies against the muscarinic-3 receptor have been found in SSc and may play a critical role in dysmotility in blocking the cholinergic neurotransmission and inhibition of acetylcholine action at smooth muscle cells [16, 37, 38]. In addition, altered TGF-β signaling may be related to spontaneous fibrosis and subsequent dysmotility in mouse colon [39].

A limitation of this study was the quality of the autopsy material. The mucosa was not assessable due to autolysis, but the other structures of the gut wall were still present. Some tissue sections could only partly be analyzed, because of improper tissue orientation. Limited tissue samples were used, which could have been resulted in missed histological abnormalities elsewhere in the intestines. Although we studied a large patient group for a rare disease as SSc, for statistical analysis the groups were small. Nevertheless, this is the first systematic study in the small bowel and colon using international guidelines for histological evaluation of the bowel wall, providing new insights in the complexity of the disease. The use of antibodies for (semi)quantification allows for more complete histological evaluation of neuronal damage. Neurons and nerve fibers were studied with HuC/D and S100, respectively, resulting in the first data using antibodies for SSc. Relevant information about clinical symptoms of intestinal hypomotility may have been missed, because of the retrospective character of our study. Currently, more clinical data are reported in the patient dossiers than for example 20 years ago, which could have caused underestimation of intestinal symptoms. Future studies should include tissues of SSc patients with known intestinal dysmotility, to combine histological features with clinical symptoms. In addition, research into the presence of autoantibodies in the (human) ENS and smooth muscle may provide new insights into involvement of these important structures for gut motility. Use of bowel resection material instead of autopsy material is recommended to improve tissue quality.

## Conclusions

In short, the postulated sequential processes leading to intestinal dysmotility in SSc are vascular damage, neuronal dysfunction, smooth muscle atrophy, and fibrosis. This sequence could not be supported by our histological findings. The histological variation between SSc patients may suggest that pathological processes are parallel rather than sequential, explaining the diversity of histological features and clinical symptoms.

## Electronic supplementary material

ESM 1 (TXT 10 kb)

## Data Availability

The datasets used and analyzed during the current study are available from the corresponding author on reasonable request.

## Abbreviations

ENS: enteric nervous system
EVG: elastica van Gieson
GI: gastrointestinal
H&E: hematoxylin eosin
ICC: interstitial cell of Cajal
PAS: periodic acid Schiff
SMA: smooth muscle actin
SSc: systemic sclerosis
